# Using Patient-Generated Health Data From Twitter to Identify, Engage, and Recruit Cancer Survivors in Clinical Trials in Los Angeles County: Evaluation of a Feasibility Study

**DOI:** 10.2196/29958

**Published:** 2021-11-26

**Authors:** Katja Reuter, Praveen Angyan, NamQuyen Le, Thomas A Buchanan

**Affiliations:** 1 Department of Public Health and Preventive Medicine The State University of New York Upstate Medical University Syracuse, NY United States; 2 Southern California Clinical and Translational Science Institute Keck School of Medicine of USC University of Southern California Los Angeles, CA United States; 3 USC Annenberg School for Communication and Journalism University of Southern California Los Angeles, CA United States; 4 Department of Medicine Keck School of Medicine University of Southern California Los Angeles, CA United States

**Keywords:** breast cancer, cancer, clinical research, clinical trial, colon cancer, infoveillance, kidney cancer, lung cancer, lymphoma, patient engagement, prostate cancer, recruitment, Twitter, social media

## Abstract

**Background:**

Failure to find and attract clinical trial participants remains a persistent barrier to clinical research. Researchers increasingly complement recruitment methods with social media–based methods. We hypothesized that user-generated data from cancer survivors and their family members and friends on the social network Twitter could be used to identify, engage, and recruit cancer survivors for cancer trials.

**Objective:**

This pilot study aims to examine the feasibility of using user-reported health data from cancer survivors and family members and friends on Twitter in Los Angeles (LA) County to enhance clinical trial recruitment. We focus on 6 cancer conditions (breast cancer, colon cancer, kidney cancer, lymphoma, lung cancer, and prostate cancer).

**Methods:**

The social media intervention involved monitoring cancer-specific posts about the 6 cancer conditions by Twitter users in LA County to identify cancer survivors and their family members and friends and contacting eligible Twitter users with information about open cancer trials at the University of Southern California (USC) Norris Comprehensive Cancer Center. We reviewed both retrospective and prospective data published by Twitter users in LA County between July 28, 2017, and November 29, 2018. The study enrolled 124 open clinical trials at USC Norris. We used descriptive statistics to report the proportion of Twitter users who were identified, engaged, and enrolled.

**Results:**

We analyzed 107,424 Twitter posts in English by 25,032 unique Twitter users in LA County for the 6 cancer conditions. We identified and contacted 1.73% (434/25,032) of eligible Twitter users (127/434, 29.3% cancer survivors; 305/434, 70.3% family members and friends; and 2/434, 0.5% Twitter users were excluded). Of them, 51.4% (223/434) were female and approximately one-third were male. About one-fifth were people of color, whereas most of them were White. Approximately one-fifth (85/434, 19.6%) engaged with the outreach messages (cancer survivors: 33/85, 38% and family members and friends: 52/85, 61%). Of those who engaged with the messages, one-fourth were male, the majority were female, and approximately one-fifth were people of color, whereas the majority were White. Approximately 12% (10/85) of the contacted users requested more information and 40% (4/10) set up a prescreening. Two eligible candidates were transferred to USC Norris for further screening, but neither was enrolled.

**Conclusions:**

Our findings demonstrate the potential of identifying and engaging cancer survivors and their family members and friends on Twitter. Optimization of downstream recruitment efforts such as screening for *digital populations* on social media may be required. Future research could test the feasibility of the approach for other diseases, locations, languages, social media platforms, and types of research involvement (eg, survey research). Computer science methods could help to scale up the analysis of larger data sets to support more rigorous testing of the intervention.

**Trial Registration:**

ClinicalTrials.gov NCT03408561; https://clinicaltrials.gov/ct2/show/NCT03408561

## Introduction

### Background

Despite significant efforts to systematically describe barriers to identifying and enrolling clinical trial participants [1,2], insufficient recruitment of study participants remains a persistent roadblock to successful clinical research and medical progress [3-8]. A systematic review of randomized controlled trials found that 76.1% (131/172) of trials discontinued because of poor recruitment [9]. As many as 86% of clinical trials do not achieve accrual targets within their specified time [2,10]. Failure to find and attract eligible participants is a key factor contributing to clinical trial recruitment issues [9].

In recent years, researchers have increasingly complemented traditional recruitment methods (eg, flyers, public posters, advertisements in newspapers, radio, and television) with social media–based approaches [11,12]. Most of these studies have used either paid advertisements or organic, nonpaid messaging strategies to recruit study participants on social media. Social media also offers publicly accessible data from those who interact with and post on these platforms. This type of user-generated data can be used to rapidly capture and describe health-related attitudes and behaviors. Self-reported data from patients are referred to as patient-generated health data, that is, “health-related data created, gathered, or inferred by or from patients and for which the patient controls data collection and data sharing” [13].

### Objectives

The goal of this pilot study is to examine the feasibility of using local user-reported data from cancer survivors and their family members and friends on the social network Twitter in Los Angeles (LA) County as a tool to enhance clinical trial recruitment at a comprehensive cancer center. According to the National Cancer Institute, “a person is considered a survivor from the time of diagnosis until the end of life” [14]. In this study, we included family members and friends because they play a critical role in caring for cancer survivors and in making cancer care decisions [15,16].

We used user-generated health data from the social network Twitter for 2 reasons: first, research has demonstrated the active use of Twitter among members of different patient communities who share their disease experiences, for example, cancer survivors [17-21], patients with diabetes [22], people with autism [23], and people with psoriasis [24]. Second, health surveillance researchers have demonstrated the usefulness of public Twitter data to understand public and patient perspectives on a range of diseases and health topics, such as COVID-19, influenza, schizophrenia, smoking, HIV, and patient safety [25-32]. In some cases, social media user data have also demonstrated a correlation between disease prevalence and the frequency with which Twitter users discussed a disease [33].

This study focused on cancer survivors and their family members and friends who discussed any of the following 6 cancer conditions on Twitter in LA County (breast cancer, colon cancer, kidney cancer, lymphoma, non–small-cell lung cancer, and prostate cancer). We hypothesized that their user-generated data could be used to identify, engage, and potentially recruit cancer survivors for cancer trials.

## Methods

### Ethical Approval

This study relied on publicly available Twitter data. The authors adhered to Twitter’s terms of service and privacy policy [34,35]. Study-related data were collected using REDCap (Research Electronic Data Capture; Vanderbilt University) at the University of Southern California (USC), a secure, web-based application designed to support data capture for research studies. Any examples of Twitter account descriptions or tweets included in this report have been paraphrased to ensure user privacy. Study approval was obtained from the Clinical Investigations Committee at the USC Norris Comprehensive Cancer Center (USC Norris; Protocol 0S-17-7) and the Institutional Review Board at USC (Protocol HS-17-00811). This study was also registered at ClinicalTrials.gov (NCT03408561).

### General Study Design and Study Setting

We set up the study as an interrupted time series with a before-and-after social media intervention.

The implementation site was USC Norris. Twitter data monitoring and data analysis were carried out at the School of Medicine at USC. The study protocol was published in the *Journal of Medical Internet Research Protocols* [36].

### Intervention

#### Overview

The social media intervention to be tested in this study involved 2 steps: (1) monitoring cancer-specific posts about 6 cancer conditions (breast cancer, colon cancer, kidney cancer, lymphoma, lung cancer, and prostate cancer) posted by Twitter users in LA County with the goal of identifying cancer survivors and their family members and friends and (2) contacting eligible users via public reply on Twitter to share information about related cancer trials that were open to accrual at USC Norris.

The intervention was used to identify potential trial participants (cancer survivors either directly or indirectly through their family members and friends) for all onboarded clinical trials. We refer to this approach as *centralized trial recruitment* because we clustered the trials into cancer disease groups and promoted only 6 disease trial groups on Twitter rather than each individual trial. This approach aligned with the USC Norris internal screening and triage process where physicians and clinical research coordinators are divided into disease-specific teams and thus will consider potential trial participants for all the relevant trials in the respective disease area. We onboarded one disease trial group every 2 weeks in a randomized order. The order in which the cancer trial disease groups were onboarded in this study was shuffled randomly using a Fisher-Yates shuffle [37]. Once a clinical trial disease group was onboarded, the trials in that group remained on for the period of this study.

#### Twitter Data Collection

To access public Twitter user data, we used Symplur Signals [38], a health care social media analytics platform that maintains a database of disease- and health-related Twitter posts and user data from the Twitter application programming interface. The messages that we extracted included at least one of the identified keywords related to the 6 cancer conditions of interest. The keywords and hashtags used in the search were determined using an iterative process based on an established conceptual framework for social data collection and quality assessment [39] and the Symplur Signals disease hashtag project [38]. The complete list of keywords used in the data search is listed in Multimedia Appendix 1.

#### Twitter Data Verification and Analysis

We reviewed both retrospective and prospective data posted by Twitter users in LA County between July 28, 2017 and November 29, 2018. Because of the focus of the project, the analysis data set was limited to original tweets. Retweets (ie, Twitter posts shared by users who did not compose the original tweet) were removed from the data set. The goal was to identify cancer survivors and their family members and friends for each trial disease group. Twitter accounts and posts were reviewed and coded by 3 independent team members (KR, NL, and Alicia MacLennan). The coprincipal investigator reviewed any discrepancies to help resolve instances of disagreement between the coders.

We used a hybrid approach of qualitative research and machine learning (ML) methods to reliably determine the Twitter accounts of cancer survivors and their family members and friends. First, the classification of cancer survivors was based on self-reported information by Twitter users in either their profile description or tweet content. Data from users who did not clearly state their cancer survivor status (or the cancer survivor status of a family member or friend) were excluded from the study. To do so, we manually reviewed each Twitter account (N=25,032), including the Twitter profile description (paraphrased example of a Twitter profile description of a cancer survivor: *Hard working man with big dreams, cancer survivor*) and the content from the tweets in our data set (paraphrased example of tweet from a family member of a cancer survivor: *Here’s an update from my sister about her fight with stage IV colorectal cancer).* Only those data were included in the final data set in which users (cancer survivors or family members and friends) clearly mentioned their cancer experience or survivorship status (eg, on or off treatment). During the data review, we excluded non-English tweets from the analysis data set. In addition, 2 research team members independently reviewed the user accounts and coded the demographic characteristics of the Twitter users. We used 3 types of data for this analysis: (1) user profile description, (2) username, and (3) profile picture. Cases which were not clear were coded *unclear*. Because of the limited demographic information on Twitter, we limited this analysis to sex and race (people of color vs White).

Second, we used the ML program Brontometer (formerly BotOrNot) [40] established by Indiana University to verify our selection of human accounts. The program identifies automated Twitter accounts, the so-called bots [41], created by industry and interest groups that influence discussions and promote specific ideas or products [42,43]. Messages from these accounts pollute social and health research data sets. Botometer examines multiple variables such as the account’s network (diffusion patterns), user (metadata), friends (account’s contacts), temporal patterns (tweet rate), and sentiment (content of messages) and detects automated accounts with a 95% success rate [40].

### Eligibility

#### Characteristics of Eligible Clinical Trials

Clinical trials were required to meet the eligibility criteria presented in Textbox 1. Trial selection was independent of cancer stage. The 6 cancer trial disease categories were selected based on 2 factors: the results of a preliminary Twitter data analysis in California to determine the most frequently mentioned cancer topics in the region and the number of clinical trials at USC Norris that were open for accrual at the time. Between January 1, 2016 and January 30, 2017, we found 36,502 Twitter users in California who had sent a total of 159,396 Twitter messages in English including at least one of the selected 6 cancer disease terms (unpublished data from Symplur Signals). A preliminary analysis of clinical trials at USC Norris between January 1, 2017 and July 7, 2017 identified 84 clinical trials that were open for accrual at the time and were eligible for this study. We intended to onboard all eligible trials in the 6 cancer disease areas that were open for accrual at the time of the onset of this study.

**Inclusion and exclusion criteria for the clinical trials included in this study.**

**Inclusion criteria**

1. Focus is on one of the 6 cancer disease types: breast cancer, colon cancer, kidney cancer, lymphoma, non–small-cell lung cancer, or prostate cancer.
2. Is Institutional Review Board–approved and open to accrual at the University of Southern California Norris Comprehensive Cancer Center.
3. Is a phase 1 trial in expansion, phase 2 or 3.
4. Is an interventional trial.
5. Has recruited in English
6. Has recruited for at least 9 months at enrollment.
7. Has set a monthly accrual target of ≥1 and annual accrual target of ≥12.

**Exclusion criteria**

- Is a phase 1 trial in dose escalation.

#### Characteristics of Eligible Twitter Users

**Figure 1:**
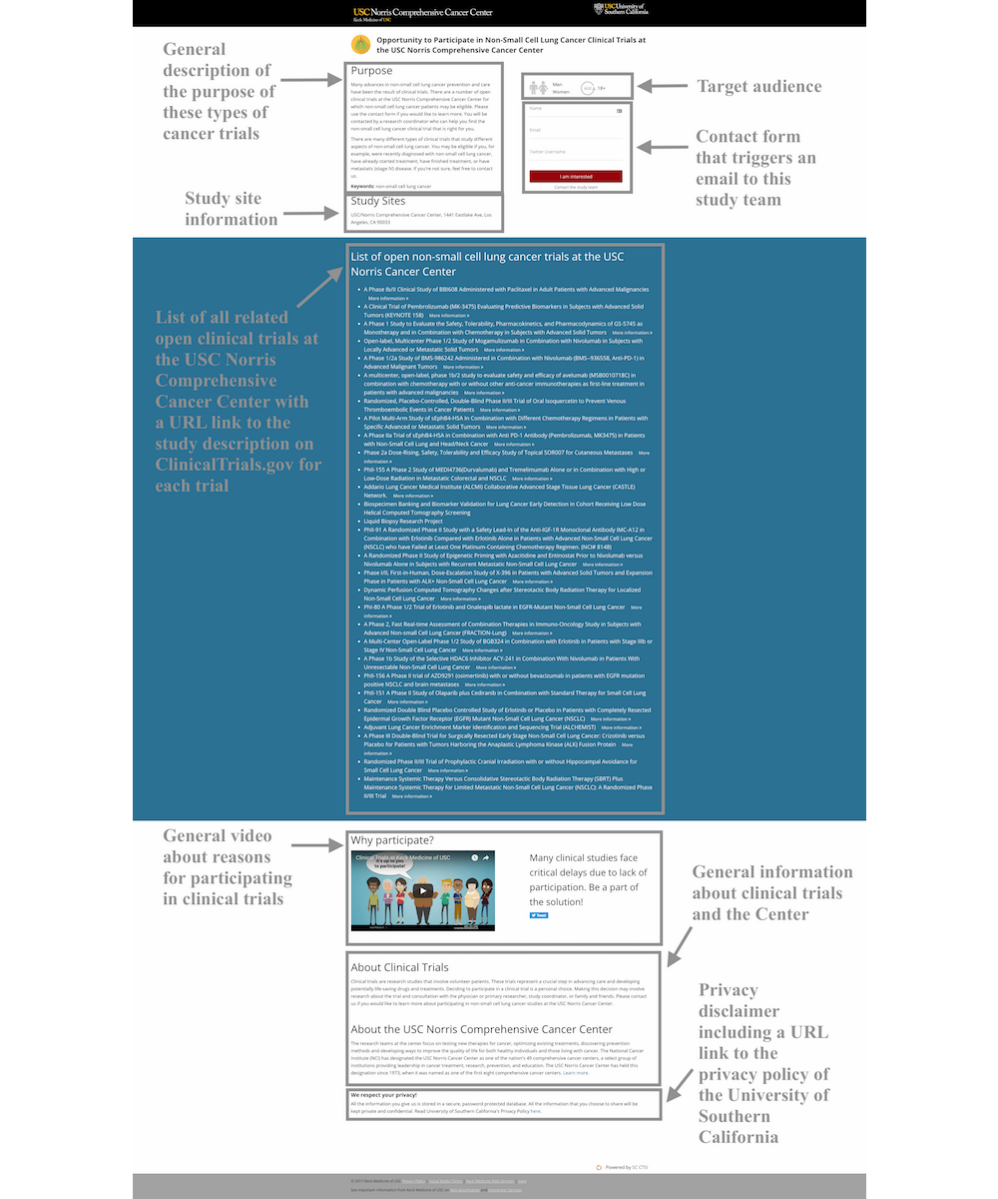
Example of a webpage that includes information about the clinical trials on lung cancer that are open to accrual at the University of Southern California (USC) Norris Comprehensive Cancer Center. Squares highlight the following page elements: a general video about reasons for participating in clinical trials, general information about clinical trials and USC Norris, and a privacy disclaimer including a URL link to the privacy policy of the USC. USC: University of Southern California.

Participant recruitment for the onboarded clinical trials occurred on the social network Twitter. The study was limited to those Twitter users who met the eligibility criteria outlined in Textbox 2. We applied both Boolean and Regex location code categories (Multimedia Appendix 2) to determine the user locations. Any Twitter user located in LA County who mentioned at least one of the selected cancer disease topics (Multimedia Appendix 1) was contacted via Twitter using the public reply feature. We included all potential trial participants in this study who expressed interest in trial participation via Twitter or through the contact form on the trial webpage (Figure 1). They were invited to an initial phone prescreening.

**Inclusion and exclusion criteria for identifying eligible Twitter users.**

**Inclusion criteria**

1. Is located in Los Angeles County based on the self-reported description provided on user’s Twitter profile.
2. Mentions in any of their Twitter messages at least one word or hashtag related to the 6 cancer disease types.
3. Message is an original Twitter message or reply to another user’s message
4. Message indicates that Twitter user has been diagnosed with cancer or that they know someone who has been diagnosed with cancer.

**Exclusion criteria**

1. Is younger than 18 years.
2. Notes that a relative or friend has died of the disease.
3. Retweets (ie, user shares message that other Twitter users sent).
4. Is a cancer survivor in remission with reduced signs and symptoms of the cancer.
5. Is a cancer survivor without any traces of cancer.

#### Recruitment of Eligible Twitter Users

Any Twitter user who met the eligibility criteria (Textbox 2) was contacted via Twitter through the public message reply feature. No advertised (paid) messages were used in the outreach. The outreach messages (Textbox 3) were sent to each user via the @USCTrials Twitter account. Each of the 3 messages served a different purpose in building trust and fostering investigator transparency [36,44]. The first message included a link to the related study webpage (Figure 1) that provided more information and allowed interested users to contact the trial coordination team.

**Outreach messages used to contact Twitter users.**

**Message type and the message**

- Reply to the user’s post that mentions the cancer disease including the link to related study webpage: “Dear (name): we noticed your mention of #kidneycancer and wanted to reach out. Did you know about these open #disease studies @KeckMedUSC? You can find more information here: (study page link) #ClinicalTrial”
- Message to introduce the research project: “We are reaching out to you as part of a research project trying to understand if Twitter can be used to better connect patients with clinical research opportunities.”
- Privacy and security disclaimer: “The security of social media is not guaranteed. Contact us about the study. Do not post if concerned about privacy.”

### Participant Compensation

Participants (ie, contacted Twitter users) did not receive monetary compensation for their participation in the social media–based outreach but could receive compensation if they consented to participate in one of the clinical trials, depending on the trial.

### Outcome Measures

The primary study outcomes included the proportion of Twitter users who (1) were identified on Twitter; (2) engaged with outreach messages through Twitter replies, mentions, likes, retweets, direct messages, or contact form use on the trial webpage; and (3) enrolled in a cancer trial. This paper reports the results of the tested intervention and related feasibility outcomes. It does not include the qualitative study results, that is, results of the qualitative interviews with study coordinators at the Cancer Center, to assess the feasibility and the level of acceptance that was also described in the protocol paper [36].

### Analysis

#### Twitter Data Coding

All coding was performed independently by at least 2 research team members. The Cohen κ coefficient [45,46] was used to assess the interrater agreement of the 3 coders who analyzed the Twitter accounts and posts. A coefficient greater than 0.8 was considered substantial for this study.

#### Statistical Analysis

As this was a pilot study, *P* values were of limited use to determine group differences. We used descriptive statistics to describe our study findings and calculated the proportions of targeted Twitter users who were identified, engaged with outreach messages, and enrolled in cancer trials.

### Data Sharing

The deidentified and aggregated data that support the findings of this study are available in the data repository figshare [47-49].

## Results

### Volume of Cancer-Related Conversations on Twitter in LA County

**Figure 2:**
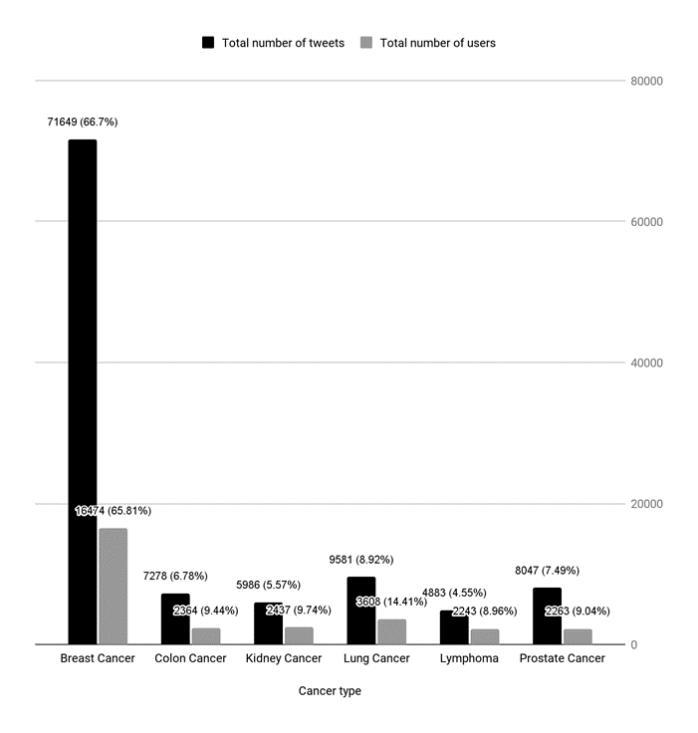
Number of Twitter posts and users in Los Angeles County by cancer type found between July 28, 2017, and November 29, 2018 in the data set of 107,424 posts.

Between July 28, 2017, and November 29, 2018, we retrieved and analyzed 107,424 tweets in English posted by 25,032 unique Twitter users in LA County (Figure 2). We found user-generated posts for all 6 cancer conditions. The highest number of tweets (71,649/107,424, 66.7%) and the highest number of users (16,474/25,032, 65.81%) were related to breast cancer (Figure 2). The topic lymphoma resulted in the lowest number of tweets (4883/107,424, 4.55%) and the lowest number of users (2243/25,032, 8.96%).

### Cancer Survivors and Their Family Members and Friends on Twitter in LA County

Of the 25,032 unique Twitter users in LA County who mentioned any of the cancer conditions, we identified 434 (1.73%) as eligible cancer survivors or their family members and friends. Approximately one-third (127/434, 29.3%) of them were cancer survivors, whereas the majority (305/434, 70.3%) mentioned a family member or friend who had cancer. Less than (2/434, 0.5%) discussed their own cancer experience and the cancer of a family member or friend. In (4/434, 0.9%) of the posts, the person affected by cancer was unclear. Of the 434 users, 175 (40.3%) discussed breast cancer, 86 (19.8%) lymphoma, 64 (14.7%) lung cancer, 61 (14.1%) colon cancer, 28 (6.5%) prostate cancer, and 20 (4.6%) kidney cancer.

We further attempted to determine the sex and race of the eligible Twitter users (Table 1) and found that half of them were female, about one-third were male, and the sex of less than one-fifth was unclear. More than half of them were White, and about one-fifth were people of color.

**Table 1:** Demographics of the Twitter users who were identified as either cancer survivors or their family members and friends (n=434).

Characteristics			Participants, n (%)
**Sex**			
	Male	140 (32.3)	
	Female	223 (51.4)	
	Unclear	71 (16.4)	
**Race**			
	People of color	99 (22.8)	
	White	254 (58.5)	
	Unclear	81 (18.7)	

### Responses to Cancer Trial Recruitment Messages on Twitter

**Figure 3:**
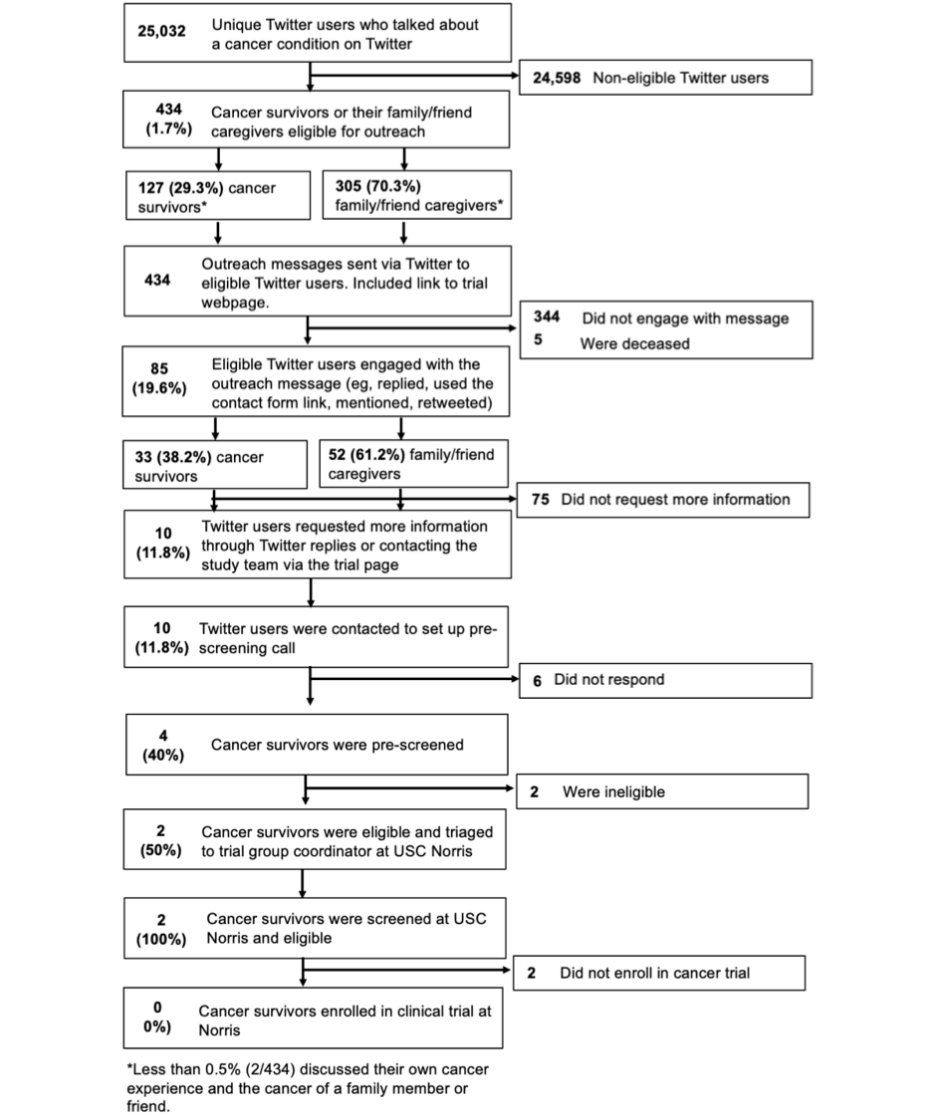
Results for the main study procedures. USC Norris: University of Southern California Norris Comprehensive Cancer Center.

Of the 434 targeted Twitter users we identified as eligible cancer survivors or their family members and friends, 85 (19.6%) engaged with the outreach messages (cancer survivors: 33/85, 38% and family members and friends: 52/85, 61%). We defined message engagement as any of the following user actions (on Twitter: reply, mention, *like*, retweet, and direct message and on the study webpage: contact form used to get in touch with the trial coordinator). In addition, of the 434 targeted Twitter users, 7 (1.6%) blocked the project account @USCTrials after the outreach, and 5 (1.2%) were deceased (based on replies by relatives or friends on Twitter). Figure 3 presents the results of the main study procedures.

Positive user engagement regarding the intervention included feedback (paraphrased) such as:

I just finished my treatment, but this is such a good use of social media.

Do you know of programs or trials for colon cancer patients? I would like to learn if my family member is eligible for trials in the area.

However, it also became evident that some users included detailed medical information in their responses that posed user privacy risks, for example (paraphrased):

My wife is stage 4. They found lepto mets in her brain. After inserting an ommaya reservoir they started intrathecal chemotherapy, which didn’t work so now they are considering partial brain radiation. She was also on letrozole and ibrance but they took her off due to her low blood counts. We don’t favor brain radiation and would like another option. Do you have any related clinical trials?

Engagement with outreach messages varied according to cancer type. We found the highest level of engagement with the outreach messages for breast cancer (26/85, 30%), followed by lymphoma (22/85, 25%), lung cancer (18/85, 21%), colon cancer (12/85, 14%), prostate cancer (4/85, 4%), and kidney cancer (3/85, 3%).

We attempted to describe the characteristics of the 85 users who engaged with the recruitment messages. A quarter of them were male, whereas the majority were female. About one-fifth were people of color, whereas the majority were White (Table 2).

**Figure 4:**
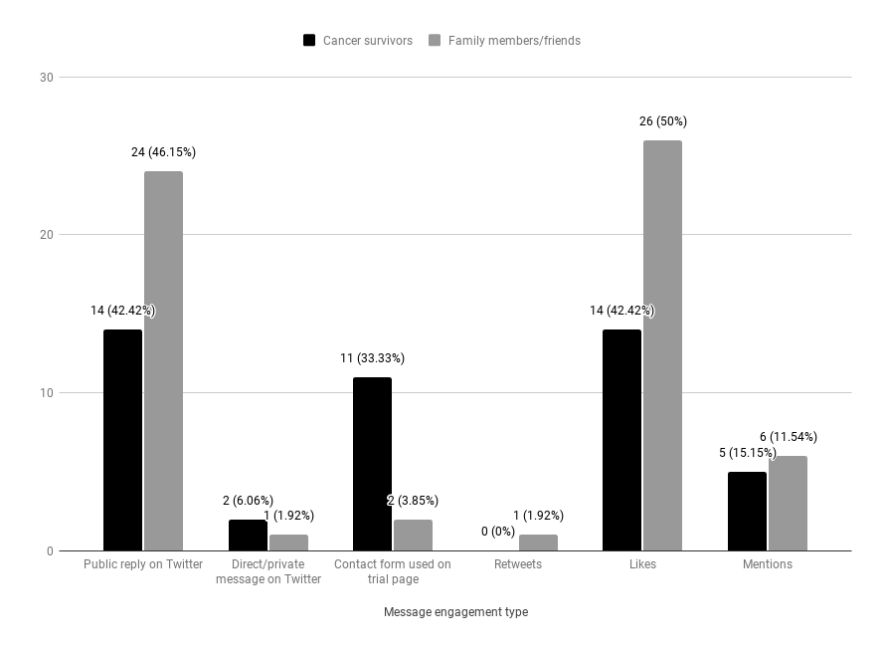
Message engagement with outreach messages by cancer survivors versus family members and friends.

We further examined the engagement type with outreach messages by cancer survivors versus family members and friends (Figure 4). Of the 85 users who engaged with outreach messages, 33 (38%) were cancer survivors and 52 (61%) were family members and friends. Public replies (14/33, 42%), contact form use (11/33, 33%), and *likes* (14/33, 42%) were the primary forms of engagement among cancer survivors, whereas family members and friends primarily engaged via public reply (24/52, 46%) and *likes* (26/52, 50%).

**Table 2:** Demographics of the Twitter users who engaged with the outreach messages (n=85).

Characteristics			Participants, n (%)
**Sex**			
	Male	22 (25)	
	Female	52 (61)	
	Unclear	11 (12)	
**Race**			
	People of color	16 (18)	
	White	57 (67)	
	Unclear	12 (14)	

### Prescreening, Screening, and Enrollment

Of the 85 Twitter users who engaged with the outreach messages, 10 (11%) requested more information through replies on Twitter, through emails, or through the use of the contact form on the trial webpage. Paraphrased example responses are as follows:

Thank you for sharing this information. How do I apply? I did not know about this work at USC.

I was diagnosed with colon cancer (stage 3) and had my operation in December. Now I’m on infusion chemotherapy treatment, xelox and capecetabine. Please call me; I’m available by phone.

Of the 10 individuals who requested more information, 1 (10%) had breast cancer; 5 (50%), colon cancer; 1 (10%), kidney cancer; and 3 (30%), lymphoma. We followed up with these users either on Twitter or via email (in the case a user provided email information) to set up a prescreening phone call, but we only managed to set up a prescreening call with 40% (4/10) of them.

We triaged 2 eligible candidates—one with colon cancer and one with kidney cancer—and referred them to the USC Norris coordinator team for further screening. One participant qualified for kidney cancer clinical trials, and the other qualified for colon cancer clinical trials. Finally, regardless of the abovementioned engagement with outreach messages, none of the recruitment outreach and engagement among Twitter users resulted in patients enrolling in a trial.

## Discussion

### Principal Findings

Our findings demonstrate that both cancer survivors and their family members and friends affected by different cancers can be found on Twitter based on their social media activity (ie, posts about cancer). In this study, approximately one-third of the identified Twitter users were cancer survivors who discussed their cancer experience, whereas others were family members and friends. Our data support the findings of previous studies that showed that both groups were present on social media for various reasons, such as seeking information resources and emotional and peer support on the web [17-21,50-52].

We found differences in the volume of posts and users across the 6 monitored cancer types. These variations could be explained by cancer prevalence and the size of the respective disease community on Twitter. For example, breast cancer is the most common type of cancer among women in the United States [53] and the third most common type in LA County [54]. It was also the most prevalent cancer topic in our data set (40.3% of the posts).

Our data further indicate that diverse, non-White cancer survivors and family members and friends can be found on social media, although more than half of the identified users were White. However, the social media user base has grown more representative of the broader population, and this trend is expected to continue. According to Pew Research [55], the percentage of American adults who use at least one social media site has increased across age groups: for example, among 18-29-year-olds, from 78% in 2009 to 90% in 2019; and among 50-64-year-olds, from 25% in 2009 to 69% in 2019. The same trend was reported for the racial diversity of social media users, but it may depend on the location in the United States. For example, based on data from 2019, on Twitter, 25% of the users are Hispanic, 24% Black, and 21% White.

Of the 434 targeted Twitter users we identified as eligible cancer survivors or their family members and friends, 85 (19.6%) engaged with the outreach messages. Some users included detailed medical information in their responses, which posed user privacy risks. Future studies are needed to develop strategies for managing such responses. We encouraged these Twitter users to delete their Twitter responses and continue the conversation with our team via the private direct message feature on Twitter, email, or phone.

Because of a lack of baseline data, it is challenging to determine whether we should have expected a higher message engagement rate. We directly contacted the cancer survivors or their family members and friends on Twitter who had mentioned the cancer keywords we were monitoring. We acknowledge that the level of acceptance of our direct targeting approach might vary among social media users. Gelinas et al [44] described this form of recruitment as *active*, which “occurs when research staff approach and interact with specific individuals with the aim of enrolling them in research, usually on the basis of knowledge of characteristics that would make them suitable candidates for particular trials” [44]. However, before we implemented this pilot study, we examined the level of concern among Twitter users and nonusers about using Twitter surveillance data to recruit potential clinical trial participants [56]. We found that most Twitter users did not consider monitoring Twitter for clinical trial recruitment as inappropriate surveillance or a violation of privacy. That said, the expressed attitudes were highly contextual, depending on factors such as the type of disease or health topic (eg, HIV vs cancer vs smoking), the entity or person monitoring users on Twitter, and the monitored information. We found that the monitoring of Twitter user data related to HIV raised the highest level of concern compared with monitoring related to cancer, HPV, obesity, or smoking. This may be partly because HIV is still associated with stigma [57]. Bender et al [58] suggested that “within health information, there are gradients of sensitivity,” and certain health topics and disease types, such as cancer, may be considered less sensitive personal health information.

Furthermore, we did not tailor the content and language of the outreach messages to specific sex or racial and ethnic groups. Doing so may aid in engaging diverse social media users. One-fourth of those engaged with outreach messages were male, whereas more than half were female. About one-fifth were people of color, whereas more than half were White. Future research could assess the effectiveness of culturally tailored messaging strategies for this type of active recruitment.

Although one-fifth of the targeted Twitter users engaged with the outreach messages, our team was challenged to translate this type of user attention into prescreening phone calls, screening, and ultimate trial enrollment. We were cautiously optimistic that we would be able to enroll trial participants using this method. Our goal was to establish an effect size using the pilot data. However, we did not complete any enrollment. Nonetheless, we believe that the identification of cancer survivors and family members and friends creates opportunities for study recruitment and intervention research. Future research should examine the barriers to social media–based clinical trial recruitment.

The fact that less than half of the identified Twitter users responded to our efforts to set up a phone call indicates that successful social media engagement may not correlate with clinical trial enrollment. Without additional research into the barriers to social media–based clinical trial recruitment, it is difficult to identify adjustments required for the study design. Inputs from social media–based patient communities could shed light on the issue of the significant drop-off.

The barriers to follow through are still unknown and need to be studied. The lack of follow-through could be because of the variables that affect downstream clinical trial processes, such as screening and consenting. These aspects of the recruitment process are unrelated to social media monitoring and outreach. In addition, barriers can be at the patient level (eg, education about clinical trials and financial barriers), at the system level (eg, inability to take time off work to schedule calls or appointments), or even at the community level (eg, lack of public transportation to the site and lack of resources) and can include factors related to the social determinants of health [7,59]. Approaches such as patient navigation have proven to be effective in addressing barriers at multiple levels in other settings [60] and could be adapted for social media–based recruitment methods. Digital forms of patient navigation could employ digital disease community members, influencers, and disease advocates on social media. We propose virtual focus groups and in-person interviews with cancer survivors on social media to identify barriers and facilitators of digital trial recruitment efforts and other types of interventions.

A detailed cost-effectiveness analysis was not included in this pilot study. However, information about the time and staff required may prove helpful in informing similar surveillance recruitment efforts. In this 1-year pilot, 4 team members were involved (10%-50% effort) to plan and implement the study (eg, developing the search strategy and weekly searches, coding extracted tweets and users to determine cancer survivors and family members and friends, sending outreach messages, tracking responses, following up and prescreening, and coordinating with the Cancer Center team). Future studies could conduct more robust cost-effectiveness and sustainability analyses.

### Limitations and Challenges

We recognize that this is a pilot study and that the generalizability of the results is somewhat limited because cancer-related Twitter messages from locations outside of LA County and non-English messages (eg, Spanish) were not included. Moreover, social media interventions favor those with internet access and exclude those without, thus introducing bias in the participant population. The social media user base on Twitter is generally younger (38% are aged 18-29 years), college-educated, and located in urban or suburban areas [53] compared with the *average* study participant. Thus, recruiting via Twitter has the potential to select for this segment of the population and could therefore introduce bias. Future research should determine the extension of the findings and conclusions to the population at large.

Much of the Twitter data we used were prospective. However, we also included retrospective social media data (ie, relevant Twitter messages sent by users in LA County 6 months before the onset of the study). Whether a user’s message was posted 1 week or 6 months ago may have affected the user’s engagement with the outreach messages. It is also possible that factors such as disease awareness months and trending news affect the attention to outreach messages.

Finally, the classification of cancer survivors was based on self-reported information from Twitter users. Although we excluded any data from users who did not clearly state their cancer survivor status (or the cancer survivor status of a family member or friend), there is a risk of misclassification, particularly if users provide false information. However, we are not aware of any research that suggests *patient imposters* on social media, that is, individuals falsely posing as patients.

### Conclusions

Our findings demonstrate the potential of identifying and engaging cancer survivors and their family members and friends on Twitter, but we were unable to enroll clinical trial participants. However, we believe that the identification of cancer survivors and family members and friends creates opportunities for study recruitment and intervention research. Future research could examine barriers to this type of social media–based clinical trial recruitment, ways to tailor efforts downstream of the initial engagement, such as prescreening, screening, and consenting to the preferences of *digital populations* on social media, and the feasibility of the approach for other diseases, locations, languages, and social media platforms. Furthermore, the integration of computer science approaches such as deep learning, ML, and natural language processing to scale up the analysis of larger data sets would further support more rigorous testing of the intervention.

## Appendix

Multimedia Appendix 1 Cancer-specific keywords and hashtags were used to monitor Twitter user conversations in Los Angeles County.

Multimedia Appendix 2 Boolean and Regex location code categories were used in this study to determine a user’s location in Los Angeles County.

## Abbreviations

LA: Los Angeles
ML: machine learning
REDCap: Research Electronic Data Capture
USC: University of Southern California

## References

[1] Ross S, Grant A, Counsell C, Gillespie W, Russell I, Prescott R (1999). Barriers to participation in randomised controlled trials: a systematic review. J Clin Epidemiol.

[2] Treweek S, Lockhart P, Pitkethly M, Cook JA, Kjeldstrøm M, Johansen M, Taskila TK, Sullivan FM, Wilson S, Jackson C, Jones R, Mitchell ED (2013). Methods to improve recruitment to randomised controlled trials: Cochrane systematic review and meta-analysis. BMJ Open.

[3] Treweek S, Pitkethly M, Cook J, Fraser C, Mitchell E, Sullivan F, Jackson C, Taskila TK, Gardner H (2018). Strategies to improve recruitment to randomised trials. Cochrane Database Syst Rev.

[4] Bower P, Brueton V, Gamble C, Treweek S, Smith CT, Young B, Williamson P (2014). Interventions to improve recruitment and retention in clinical trials: a survey and workshop to assess current practice and future priorities. Trials.

[5] Institute of Medicine (2012). Envisioning a Transformed Clinical Trials Enterprise in the United States: Establishing an Agenda for 2020: Workshop Summary.

[6] Institute of Medicine (2012). Public Engagement and Clinical Trials: New Models and Disruptive Technologies: Workshop Summary.

[7] Nipp RD, Hong K, Paskett ED (2019). Overcoming barriers to clinical trial enrollment. Am Soc Clin Oncol Educ Book.

[8] Huang GD, Bull J, Johnston McKee K, Mahon E, Harper B, Roberts JN, CTTI Recruitment Project Team (2018). Clinical trials recruitment planning: a proposed framework from the Clinical Trials Transformation Initiative. Contemp Clin Trials.

[9] Briel M, Olu KK, von Elm E, Kasenda B, Alturki R, Agarwal A, Bhatnagar N, Schandelmaier S (2016). A systematic review of discontinued trials suggested that most reasons for recruitment failure were preventable. J Clin Epidemiol.

[10] Lamberti MJ, Mathias A, Myles JE, Howe D, Getz K (2012). Evaluating the impact of patient recruitment and retention practices. Drug Information J.

[11] Darmawan I, Bakker C, Brockman TA, Patten CA, Eder M (2020). The role of social media in enhancing clinical trial recruitment: scoping review. J Med Internet Res.

[12] Topolovec-Vranic J, Natarajan K (2016). The use of social media in recruitment for medical research studies: a scoping review. J Med Internet Res.

[13] Demiris G, Iribarren SJ, Sward K, Lee S, Yang R (2019). Patient generated health data use in clinical practice: a systematic review. Nurs Outlook.

[14] (2011). NCI dictionary of cancer terms. National Cancer Institute.

[15] Kim Y, Kashy DA, Kaw CK, Smith T, Spillers RL (2009). Sampling in population-based cancer caregivers research. Qual Life Res.

[16] (2010). Informal caregivers in cancer: roles, burden, and support (PDQ)–health professional version. National Cancer Institute.

[17] Modave F, Zhao Y, Krieger J, He Z, Guo Y, Huo J, Prosperi M, Bian J (2019). Understanding perceptions and attitudes in breast cancer discussions on Twitter. Stud Health Technol Inform.

[18] Sedrak MS, Salgia MM, Bergerot CD, Ashing-Giwa K, Cotta BN, Adashek JJ, Dizman N, Wong AR, Pal SK, Bergerot PG (2019). Examining public communication about kidney cancer on Twitter. JCO Clin Cancer Inform.

[19] Xu S, Markson C, Costello KL, Xing CY, Demissie K, Llanos AA (2016). Leveraging social media to promote public health knowledge: example of cancer awareness via Twitter. JMIR Public Health Surveill.

[20] Sugawara Y, Narimatsu H, Hozawa A, Shao L, Otani K, Fukao A (2012). Cancer patients on Twitter: a novel patient community on social media. BMC Res Notes.

[21] Katz MS, Utengen A, Anderson PF, Thompson MA, Attai DJ, Johnston C, Dizon DS (2016). Disease-specific hashtags for online communication about cancer care. JAMA Oncol.

[22] Karahan I, Yürekli A, Özcömert OR, Oktaş B, Çifci A (2020). Who tweets about diabetic foot on twitter and which tweets are more attractive?. Int J Low Extrem Wounds.

[23] Hswen Y, Gopaluni A, Brownstein JS, Hawkins JB (2019). Using Twitter to detect psychological characteristics of self-identified persons with autism spectrum disorder: a feasibility study. JMIR Mhealth Uhealth.

[24] Li W, Le N, Lee DJ, Reuter K (2020). Analysis of psoriasis-related posts on Twitter: an abundance of patient-driven advocacy versus a scarcity of dermatologists. J Am Acad Dermatol.

[25] Gao J, Zheng P, Jia Y, Chen H, Mao Y, Chen S, Wang Y, Fu H, Dai J (2020). Mental health problems and social media exposure during COVID-19 outbreak. PLoS One.

[26] Abd-Alrazaq A, Alhuwail D, Househ M, Hamdi M, Shah Z (2020). Top concerns of tweeters during the COVID-19 pandemic: a surveillance study. J Med Internet Res.

[27] Hswen Y, Naslund JA, Brownstein JS, Hawkins JB (2018). Monitoring online discussions about suicide among Twitter users with schizophrenia: exploratory study. JMIR Ment Health.

[28] Wagner M, Lampos V, Cox IJ, Pebody R (2018). The added value of online user-generated content in traditional methods for influenza surveillance. Sci Rep.

[29] Nielsen RC, Luengo-Oroz M, Mello MB, Paz J, Pantin C, Erkkola T (2017). Social media monitoring of discrimination and HIV testing in Brazil, 2014-2015. AIDS Behav.

[30] Malik A, Li Y, Karbasian H, Hamari J, Johri A (2019). Live, love, juul: user and content analysis of Twitter posts about juul. Am J Health Behav.

[31] Bychkov D, Young S (2018). Social media as a tool to monitor adherence to HIV antiretroviral therapy. J Clin Transl Res.

[32] Nakhasi A, Bell SG, Passarella RJ, Paul MJ, Dredze M, Pronovost PJ (2019). The potential of Twitter as a data source for patient safety. J Patient Saf.

[33] Tufts C, Polsky D, Volpp KG, Groeneveld PW, Ungar L, Merchant RM, Pelullo AP (2018). Characterizing tweet volume and content about common health conditions across pennsylvania: retrospective analysis. JMIR Public Health Surveill.

[34] Twitter Terms of Service.

[35] Twitter Privacy Policy.

[36] Reuter K, Angyan P, Le N, MacLennan A, Cole S, Bluthenthal RN, Lane CJ, El-Khoueiry AB, Buchanan TA (2018). Monitoring Twitter conversations for targeted recruitment in cancer trials in Los Angeles County: protocol for a mixed-methods pilot study. JMIR Res Protoc.

[37] Durstenfeld R (1964). Algorithm 235: Random permutation. Commun ACM.

[38] Disease hashtags. Symplur.

[39] Kim Y, Huang J, Emery S (2016). Monitoring Twitter conversations for targeted recruitment in cancer trials in Los Angeles County: protocol for a mixed-methods pilot study. J Med Internet Res.

[40] Davis C, Varol O, Ferrara E, Flammini A, Menczer F (2016). BotOrNot: a system to evaluate social bots. arXiv csSI.

[41] Ferrara E, Varol O, Davis C, Menczer F, Flammini A (2016). The rise of social bots. Commun ACM.

[42] Mønsted B, Sapieżyński P, Ferrara E, Lehmann S (2017). Evidence of complex contagion of information in social media: an experiment using Twitter bots. PLoS One.

[43] Shao C, Ciampaglia GL, Varol O, Yang K, Flammini A, Menczer F (2018). The spread of low-credibility content by social bots. Nat Commun.

[44] Gelinas L, Pierce R, Winkler S, Cohen IG, Lynch HF, Bierer BE (2017). Using social media as a research recruitment tool: ethical issues and recommendations. Am J Bioeth.

[45] Cohen J (2016). A coefficient of agreement for nominal scales. Edu Psychol Measur.

[46] McHugh ML (2012). Interrater reliability: the kappa statistic. Biochem Med (Zagreb).

[47] Reuter K (2021). Dataset: weekly tweet counts by cancer type per week. Social and Community Informatics.

[48] Reuter K (2021). Dataset: total tweet and user counts by cancer type. Social and Community Informatics.

[49] Reuter K (2021). Dataset: outreach message engagement. Social and Community Informatics.

[50] Daniels SR, Yang C, Toohey SJ, Willard VW (2021). Perspectives on social media from adolescents and young adults with cancer. J Pediatr Oncol Nurs.

[51] Warner EL, Kirchhoff AC, Wilson A, Cloyes KG, Sun Y, Waters AR, Nelson T, Ellington L (2021). Social support enactments on social media during the first 6 months of young adult cancer caregiving. J Cancer Surviv.

[52] Booth A, Bell T, Halhol S, Pan S, Welch V, Merinopoulou E, Lambrelli D, Cox A (2019). Using social media to uncover treatment experiences and decisions in patients with acute myeloid leukemia or myelodysplastic syndrome who are ineligible for intensive chemotherapy: patient-centric qualitative data analysis. J Med Internet Res.

[53] (2015). Common cancer types. National Cancer Institute.

[54] Liu L, Wang Y, Sherman R, Cockburn M, Deapen D (2016). Cancer in Los Angeles County: trends by race/ethnicity 1976-2012. Los Angeles Cancer Surveillance Program, University of Southern California.

[55] (2019). Demographics of social media users and adoption in the United States. Pew Research Center: Internet, Science & Tech.

[56] Reuter K, Zhu Y, Angyan P, Le N, Merchant AA, Zimmer M (2019). Public concern about monitoring Twitter users and their conversations to recruit for clinical trials: survey study. J Med Internet Res.

[57] Chambers LA, Rueda S, Baker DN, Wilson MG, Deutsch R, Raeifar E, Rourke SB, Stigma RT (2015). Stigma, HIV and health: a qualitative synthesis. BMC Public Health.

[58] Bender JL, Cyr AB, Arbuckle L, Ferris LE (2017). Ethics and privacy implications of using the internet and social media to recruit participants for health research: a privacy-by-design framework for online recruitment. J Med Internet Res.

[59] Marmot M (2000). Social determinants of health: from observation to policy. Med J Aust.

[60] Freeman HP (2006). Patient navigation: a community based strategy to reduce cancer disparities. J Urban Health.

