# Supplementary material for: Using Patient-Generated Health Data From Twitter to Identify, Engage, and Recruit Cancer Survivors in Clinical Trials in Los Angeles County: Evaluation of a Feasibility Study
Source: JMIR Form Res. 2021 Nov 26;5(11):e29958. doi: 10.2196/29958 (PMC8665395; doi:10.2196/29958)
Supplement: Multimedia Appendix 1 [file formative_v5i11e29958_app1.docx]

**Multimedia Appendix 1. Cancer-specific keywords and hashtags that were used for monitoring Twitter user conversations in Los Angeles County.** (Based on data from Symplur.com)

| **Cancer disease category** | **Keywords** | **Hashtags** |
| --- | --- | --- |
| Breast cancer | “Breast cancer” | #advancedbc (advanced breast cancer)  #bccww (breast cancer chat worldwide)  #bcmets (breast cancer metastatic)  #bcpea (breast cancer patient education action)  #bcsm (breast cancer social media)  #bctalk (breast cancer talk)  #bebrcaware (be BRCA mutation aware)  #brca (BRCA mutation)  #brca1 (BRCA1 gene)  #brca2 (BRCA2 gene)  #brcamutation  #breastcancer  #breastcancerawareness  #malebreastcancer  #mammogram  #mbcsm (metastatic breast cancer social media)  #metastaticbc (metastatic breast cancer)  #metastaticbreastcancer  #pinkribbon  #thinkpink  #thisismbc (this is metastatic breast cancer)  #tnbc (triple negative breast cancer)  #trackmbc (track metastatic breast cancer) |
| Colon cancer | “Colon cancer”  “Colorectal cancer”  “Bowel cancer” | #bowelcancer  #cancercolon  #coloncancer  #colonoscopy  #colorectal  #colorectalcancer  #crcsm (colorectal cancer social media)  #rectalcancer  #scopescope |
| Kidney cancer | “Kidney cancer”  Renal (for Renal Cancer and Renal Cell Carcinoma) | #kcsm (kidney cancer social media)  #kidneycancer  #renalcellcarcinoma |
| Lymphoma | Lymphoma  Hodgkins  Hodgkin’s  Hodgkin  Bloodcancer  “Blood cancer” | #bloodcancer  #bloodcancerawarenessmonth  #dlbcl (diffuse large b-cell lymphoma)  #hodgkin  #hodgkins  #lymphoma  #lymsm (lymphoma social media)  #lyphoma (frequent misspelling of lymphoma)  #nonhodgkinslymphoma  #edforumchat (annual North American Educational Forum on lymphoma) |
| Non-small cell lung cancer | “Lung cancer” | #433aday (433 a day; refers to number of people who lose their life to lung cancer every day)  #beatlungcancer  #changelc (change lung cancer)  #chatnsclc (chat non-small cell lung cancer)  #endlungcancer  #freetobreathe  #lccaregiver (lung cancer caregiver)  #lcsm (lung cancer social media)  #ldct (low dose computed tomograph; lung cancer screening for high risk patients)  #lungcancer  #lungcancerawareness  #lungcancerscreening  #nsclc (non-small cell lung cancer)  #talklungcancer |
| Prostate cancer | “Prostate” and word filter “cancer” | #movember (Movember is an annual event involving the growing of moustaches during the month of November to raise awareness of men's health issues, such as prostate cancer, testicular cancer, and men's suicide)  #oneineight (one in eight; refers to the statistic that one in eight men will be diagnosed with prostate cancer)  #pcsm (prostate cancer social media)  #prostatecancer  #prostatecancerawareness  #prostatecare  #prostatechat  #psatest (prostate-specific antigen test; refers to screening for prostate cancer) |
